# The Long and Winding Road: A Systematic Literature Review Conceptualising Pathways for Hypertension Care and Control in Low- and Middle-Income Countries

**DOI:** 10.34172/ijhpm.2020.105

**Published:** 2020-07-18

**Authors:** Rachel Brathwaite, Eleanor Hutchinson, Martin McKee, Benjamin Palafox, Dina Balabanova

**Affiliations:** ^1^Brown School, Washington University in St Louis, St Louis, MO, USA.; ^2^Department of Global Health and Development, Faculty of Public Health and Policy, London School of Hygiene and Tropical Medicine, London, UK.; ^3^Department of Health Services Research and Policy, Faculty of Public Health and Policy, London School of Hygiene and Tropical Medicine, London, UK.

**Keywords:** Systematic Review, Hypertension Control, Healthcare Delivery, Health Systems, Pathways to Care

## Abstract

**Background:** Hypertension control is poor everywhere, especially in low- and middle-income countries (LMICs). An effective response requires understanding factors acting at each stage on the patients’ pathway through the health system from entry or first contact with the health system, through to treatment initiation and follow up. This systematic review aimed to identify barriers to and facilitators of hypertension control along this pathway and, respectively, ways to overcome or strengthen them.

**Methods:** MEDLINE, EMBASE, Global Health, CINAHL Plus, and Africa-Wide Information (1980-April 2019) were searched for studies of hypertensive adults in LMICs reporting details of at least 2 adequately described health system contacts. Data were extracted and analysed by 2 reviewers. Themes were developed using NVivo in patient-related (sociodemographic, knowledge and health beliefs, health status and co-morbidities, trade-offs), social (social relationships and traditions) and health system domains (resources and processes). Results are reported according to the Preferred Reporting Items for Systematic Reviews and Meta-Analyses (PRISMA) guidelines.

**Results:** From 2584 identified records, 30 were included in the narrative synthesis. At entry, ‘health systems resources and processes’ and ‘knowledge and beliefs about hypertension’ dominated while ‘social relations and traditions’ and ‘comorbidities’ assume greater importance subsequently, with patients making ‘trade-offs’ with family priorities during follow up. Socio-demographic factors play a role, but to a lesser extent than other factors. Context matters.

**Conclusion:** Understanding the changing barriers to hypertension control along the patient journey is necessary to develop a comprehensive and efficient response to this persisting problem.

## Background

Hypertension is the leading preventable cause of illness and premature death worldwide.^1^ It is easily diagnosed and can be controlled with relatively simple interventions. Yet it is often unrecognised. When diagnosed, it requires life-long management and patients may be unaware of the need for continuous monitoring and adherence to treatment, which can be difficult to achieve. Moreover, while diagnosis and initiation of medication usually takes place in primary care, its management involves all levels of the health system, with referral to specialists if certain complications arise. Interventions to improve care have achieved modest results, and control remains surprisingly poor in countries at of all income levels.^2^ It is increasingly recognised that to be effective, responses must cover the entire patient pathway, from initial diagnosis through to long term treatment and, hopefully, control.

###  Conceptualisation of Patient Pathway

**Figure 1:**
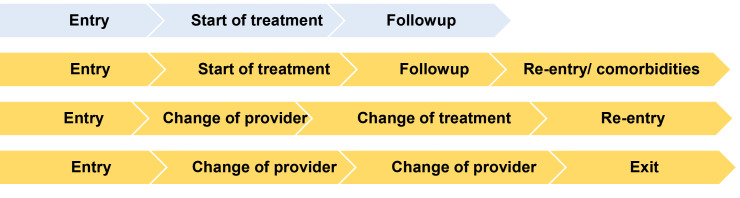
Conceptualisation of Patient Pathway for Non-communicable Disease.

Clinical guidelines typically portray the patient pathway as a linear process from diagnosis and initiation of medication to follow-up. Yet many journeys are much more complex, especially as several attempts may be needed to achieve initial control, and as hypertension is increasingly only one of several conditions affecting the patient. Figure 1 presents some archetypal pathways applicable to hypertension, and non-communicable disease in general. Which one a given patient will follow depends not only on their clinical condition but also their socio-economic characteristics, preferences, health beliefs, and features of the health system.

 Many existing guidelines divide the pathway into stages before and after initiation of treatment (‘after’ often being defined in diverse ways). They also assume, often implicitly, that once the patient is in the system, their growing familiarity with both it and their condition means that the barriers diminish. Few consider the barriers and enablers that act throughout their journey. Nor do they consider, in any detail, that the patient can interrupt, terminate or re-enter treatment at any point. Their decision to continue with treatment or not is shaped by a complex mix of knowledge, preferences, and judgements. Importantly, the factors triggering these decisions can accumulate, for example when patients face repeated long clinic waits or medicines shortages and in response seek alternative, less effective forms of care.

 This review seeks to synthesise the empirical evidence on what hampers or facilitates the patient at each stage along the pathway from entry to the health system to achieving hypertension control. This comprehensive approach fills a major gap in the literature.

## Methods

The study protocol uses the Preferred Reporting Items for Systematic Reviews and Meta-Analyses (PRISMA) guidelines and was registered with the International Prospective Register of Systematic Reviews (PROSPERO: CRD42017074786).^3^

###  Research Questions

1. How do patients with hypertension move through the healthcare system, over time and across different levels and types of care?
2. What are the barriers and enabling factors at each stage of the patient pathway? Which relate to the health system and which to patient characteristics and their families and social networks, and how?
3. How can the patient be helped to navigate the pathway successfully?

Initially, we also sought evidence on how different pathways relate to health outcomes but the necessary literature was lacking.

###  Key Concepts and Assumptions

A *Health system* comprises “all organizations, people, [resources] and actions whose primary intent is to promote, restore or maintain health [at the individual or population level].”^4^ Both supply-side factors (health systems structures and processes) and demand-side factors (patient choices) influence patient progression.

*Pathway *is understood as the patient’s progression through the health system, with the intended destination being control of hypertension without side effects and avoidance of complications.

*Point of contact *is where the patient interacts with the health system or alternative providers.

 We conceptualise the patient pathway as having 3 distinct stages, which we used to categorise the studies we included, while noting, as above, that the journey is often non-linear. The first is the initial contact with the health system (entry), perhaps associated with symptoms that may or may not be related to the diagnosis, and any decision to seek care. This includes all studies that describe diagnosis (either in a facility or during a community-based screening programme). The second, treatment initiation, begins with the first prescription and ends with being established on treatment. For those identified during community screening events, this stage also includes the moment when they contacted the formal health system and were provided with care (medicine and advice about nutritional or life-style changes), and not simply when they were diagnosed. This stage may include a referral to another level of the health system. The third is long-term management, when the patient has become established on treatment and they should be receiving follow-up by a designated provider but also including further referral, and departure and re-entry into the system (for the existing or new condition). There was, however, a fourth set of studies that do not fall within these stages but instead discuss pathways overall or in general. The distinction between stages allows us to identify common pathways through the system but overlaps between stages were common.

###  Inclusion and Exclusion Criteria

 The review included studies:

- of adults with hypertension (systolic blood pressure >140 mm Hg +/or diastolic *blood* pressure >90 mm Hg)
- from low- and middle-income countries (LMICs)
- including at least 2 reported contacts with the system or data gathered over a period when more than one contact with the health system was anticipated
- reporting empirical findings studies (qualitative or quantitative)
- of any design
- in English.

In addition, studies (mainly qualitative) were included if they elicited patient experiences that span the length of their journey, even if not fully distinguishing stages of treatment. These also included studies where patients followed unconventional routes, including self-treatment. It excluded studies:

- of subjects under 18 years, or not having hypertension
- of patients with pregnancy induced or secondary hypertension
- from high-income countries or conducted before 1980
- including data on only one contact and no information on subsequent stages (studies that asked patients to recall previous treatment stages were also excluded)
- not distinguishing clearly between any stages of treatment.

###  Search Strategy

 The search was conducted by RB in 5 databases; MEDLINE, EMBASE, Global Health, CINAHL Plus, and Africa-Wide Information, for all relevant articles published after 1980 until April 12, 2019. A combination of key words, phrases, and medical subject headings (MeSH) for the main concepts; ‘low and middle income countries,’ ‘hypertension,’ ‘continuity of care,’ and ‘epidemiological studies’ were used (see Annex for a full search strategy in MEDLINE).

###  Extraction and Critical Appraisal for Quality Assessment

 Two independent reviewers (RB, EH) reviewed all identified abstracts by title and abstract against the inclusion criteria. Full texts of those retained were then read by the reviewers. A third reviewer (DB) adjudicated disagreements on eligibility.

 The extraction template contained fields for study objectives, how hypertension was defined, study design, sample size and socio-demographic description of study population, research methods, risk of bias, country and healthcare settings (including level of the health system), description of each contact along the patient pathway, and barriers and enablers at each stage, if available. It distinguished the different contacts along the pathway and, where this was not possible, information on barriers and facilitators related to more than one contact was included.

We critically assessed the quality of included articles using standardised checklists for observational studies (STROBE, STrengthening the Reporting of OBservational studies in Epidemiology), randomised controlled trials (CONSORT, Consolidated Standards of Reporting Trials), and qualitative and mixed method research (SRQR, Standards for reporting Qualitative Research) as appropriate.^5-7^ Articles that met at least 80% of these standards were categorised as ‘high quality,’ ‘moderate quality’ if they met between 60% and 80% of relevant standards, and ‘low quality’ if they met less than 60%. Of the 30 included studies, 9 were assessed as high quality, 18 as moderate, and 3 as low quality. Data were extracted by the 2 reviewers independently and any differences were resolved by discussion with the third reviewer.

###  Analytical Strategy

**Figure 2:**
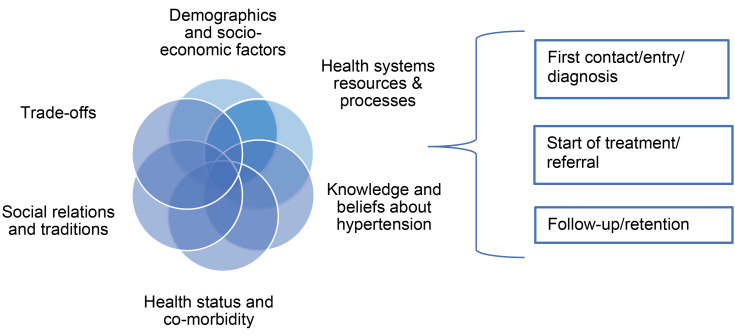
Typology of Main Domains of Barriers and Enablers Influencing Patient Pathway.

We used a mix of inductive and deductive analytical approaches. First, 2 reviewers independently thematically coded barriers and enablers of care for each of the stages described above in NVivo 11.0 (QSR International). Codes were then compared and discussed with the third reviewer and aggregated into non-exclusive categories (domains). This process of conceptualisation reflected both groupings of key themes within papers, but also codes on barriers and enablers identified from the broader literature from health systems, medical anthropology and sociology. This process was iterative; with coding followed by re-organisation of the codes, assessing their level of importance according to their prevalence and strength of evidence, followed by a further coding. This ensured that the overarching codes are distinct and represent a meaningful representation of the key barriers and enablers at different stages of hypertension care. The final typology consisted of the following 6 domains, also represented in Figure 2.

*Health systems resources and processes* included availability, accessibility and affordability of resources, such as health workers, facilities, medicines, and models of care acceptable to patients. These often assume different degrees of importance during each stage of the pathway.^8^

*Patients’ (and families) knowledge and beliefs of hypertension.* Studies of medical pluralism and syncretism find that biomedical and local or folk knowledge and beliefs about illness often interact, facilitating care or creating barriers to it and to adherence to medication.^9-11^ These often reflect how people think about their bodies over the life cycle.^12^ This domain included knowledge and beliefs about hypertension and bodies, how these may change over time, and how these may impact on adherence to formally mandated pathways.

*Health status and co-morbidities*were particularly helpful in understanding ways in which multiple co-morbidities complicate patient pathways. Given the largely asymptomatic nature of hypertension, we also considered ways in which lack of symptoms impacted on seeking treatment at all points of contact. Conversely, entering the health system in a quest for treatment of co-morbidities was sometimes a trigger to manage asymptomatic hypertension.

*Social relationships and traditions.* Drawing on medical anthropology^13-16^ and research that recognises health systems as social institutions^17,18^ we identified themes around social relationships (between the patient and their family, local community; and between the patient and health staff) impact on the patient pathway. Traditions (the association of particular foods with social events and stages in the life cycle) were coded under this domain.

*Trade-offs related to the pathways.* Seeking care in LMICs often comes at a cost, financial or otherwise, not only for the individual but their family. It often places significant burdens on family welfare. This domain was concerned with how these broader responsibilities influenced the pathway. While some studies saw this issue in terms of psychological factors (eg, forgetfulness in those with competing duties), others viewed patients as making rational trade-offs as part of their coping strategy and balancing different life and treatment decisions. Complex trade-offs made during the treatment pathway are increasingly discussed.^8^

 Given the nature of the data collected, the variation in terminology, definition of each stage a narrative synthesis was employed. Findings are structured under the three key treatment stages (entry, treatment, follow-up), and within each, grouped under the 6 domains.

## Results

###  Description of Included Studies

**Figure 3:**
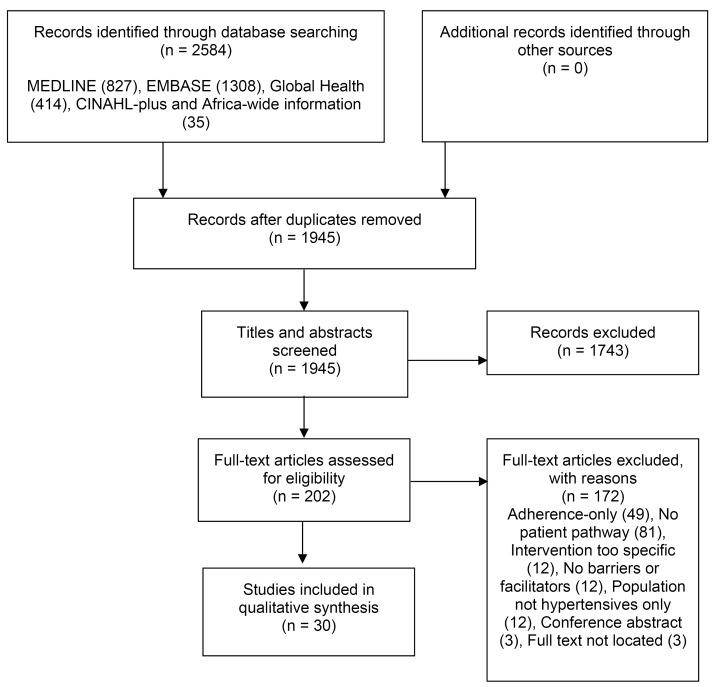
Literature Flow-PRISMA Chart. Abbreviation: PRISMA, Preferred Reporting Items for Systematic Reviews and Meta-Analyses.

The flow chart, from 1945 abstracts identified by title and abstract to the 30 included in the final synthesis, is displayed in Figure 3.

###  Description of Hypertensive Patients

The studies included, and their main characteristics, are described in Table S1 (see Supplementary file 1). Most studies were conducted in East and South-East Asia (China,^19-22^ Vietnam,^23^ Malaysia,^24^ Indonesia^25^) or sub-Saharan Africa (Ethiopia,^26^ Ghana,^27-29^ Kenya,^30-32^ Namibia,^33^ Nigeria,^34^ Tanzania,^35^ and Uganda^36^); and Egypt.^37^ Three studies were in Central and South America (Belize,^38^ Brazil,^39-41^ Colombia,^42^ Mexico^43^); 1 in South Asia (India^44^) and 1 in the Middle East (Iran^45^).

Eighteen studies were quantitative, 9 qualitative, and 3 used mixed methods. Among the quantitative studies, there were 8 prospective cohorts,^19-21,23,28,35,36,38^ three cross-sectional studies,^24,37,39^ and one prospective randomised control trial.^22^ Studies using qualitative research methods employed a mixture of semi-structured and in-depth interviews, focus group discussions, and ethnographic investigations into patients’ past experiences in seeking care for hypertension and or adherence to medication. In longitudinal studies, participants were followed up for periods from 26 days^28^ to 17 months.^23^

###  Stage 1: Entry to the Health System

Most studies examined patients recruited after initial diagnosis in primary care,^28,37,38,40,42,46,47^ followed by community screening programmes.^23,35,36^ Only 5 described facilitators and impediments to initiation of contact by patients with the health system.^37,40,42,46,47^ These fell into 2 domains: health systems, and knowledge and beliefs.

####  Health Systems Resources and Processes

The most consistent facilitator of diagnosis was the practice of checking blood pressure during attendance at primary care, which took on particular significance given that hypertension is largely symptomless until severe, and with any symptoms that are present often non-specific.^37,42,46,47^ The widespread failure to do this was linked to the high proportion of patients diagnosed at an advanced stage with symptoms, 60% of the patients in one Egyptian study.^37^ Similar findings were reported from Malaysia^46,47^ and Brazil.^40^ Many countries organise mass screening events but, as Risso-Gill and colleagues note in Malaysia, few patients subsequently attend to have their diagnosis confirmed.^46^

####  Knowledge and Beliefs

Five studies described how the combination of lack of symptoms and low awareness of its asymptomatic nature impacts on treatment seeking at the time of initial diagnosis.^42,44,46-48^ Poor understanding of the importance of treating hypertension^42,47^ also act as barriers.

###  Stage 2: Initiation of Treatment

The second stage is when patients have received a diagnosis and been advised to seek care or have been formally referred into the system from community screening events. Studies followed patients for 17 months^23^ 12 months,^35^ 6 months,^36^ and 4 months^38^ while qualitative studies interviewed patients about their overall experience but did not always specify which stage of the pathway was involved.^25,30,46^ Two reported interventions with components to improve linkage with the health system following screening.^35,36^ Linkage is a term originally used in screening for HIV, referring to establishing a link between the patient and healthcare. In Tanzania, patients were provided with information about hypertension^35^ but it had little impact on health seeking behaviour. In Uganda patients were provided with information, a voucher to cover transport costs, and a scheduled appointment,^36^ which was much more successful. In this stage, barriers and enablers related to demographics, health status, and poorly functioning health systems were most important, although differently in each context.

####  Demographics and Socio-Economic Factors

Four studies reported that older age correlated with greater propensity to seek care after diagnosis, within a community-based programme,^23^ the public sector^36,38^ and in a mix of public and private systems.^35^

Researchers explored concerns about how costs of treatment affected linking and initial attendance (see below). The negative impact of financial constraints was described in 2 qualitative studies in eastern Kenya,^30,48^ where they discouraged people from initiating care in the public sector, with a religious leader noting that “…when somebody is poor it becomes a silent killer….”

In Belize, Uganda, and Vietnam, being female was associated with an increased likelihood of seeking care after detection during screening events.^23,36,38^ However, a qualitative study in Kenya identified women’s lack of control over financial decision-making as a barrier to accessing care.^30^ This study also reported men being less likely to seek care unless experiencing severe symptoms.

Higher educational and occupational status also facilitated seeking care^36^ while in rural Uganda patients employed in manual labour (eg, farming) were more likely to link than those who were unemployed.^36^

####  Health Status and Co-morbidities

This domain highlighted the importance of co-morbidities and a family history of coronary disease. In Vietnam^23^ and Kenya^30^ behavioural risk factors for cardiovascular disease (CVD) reduced the probability of seeking care, but in Uganda, alcohol and tobacco use and were associated with increased likelihood of progressing through the health system^36^ and in Tanzania overweight patients were also more likely to seek care, although the association was only just significant.^35^ Having a family history of hypertension was a predictor of linkage to care in Uganda,^36^ but not in Vietnam.^23^ In both Vietnam and Belize, those with a personal (rather than family) history of CVD were more likely to join a hypertension programme.^23,38^ Milder hypertension independently predicted not seeking care in a community programme in Vietnam following diagnosis as did a history of other chronic diseases (explained by these patients seeking treatment elsewhere).^23^ Finally, worsening health status was considered a barrier to seeking care in Kenya,^30^ while severe hypertension facilitated joining a programme in Vietnam.^23^

The lack of symptoms associated with hypertension was identified as a barrier to seeking care following diagnosis in five studies.^25,30,35,36,48^ Naanyu proposed that gender played a role, as men are reluctant to go to a health centre unless they have a debilitating illness. One qualitative study identified forgetfulness and poor motivation as psychological barriers and personal initiative as a facilitator to linkage.^30^

####  Health System Resources and Processes

Inadequacies in the health system were identified as barriers to seeking care at health facilities. Kenyan clinics lacked staff, equipment, and medication.^30^ In Malaysia, patients referred for advice on lifestyle changes were unable to see a dietitian.^47^ Naanyu and Rachlis both described how unavailability of medicines in pharmacies and availability of herbal medicine from traditional healers diverted patients from the Kenyan public health system.^30,48^ However, in Tanzania Bovet and colleagues found this to be a very minor problem, as only one of 161 patients sought care from a traditional practitioner.^35^ However, they did find cost to be a barrier to continued attendance. In Kenya, costs of diagnosis and treatment, even in relation to public or subsidised services, pose a significant burden, and may lead to catastrophic expenditure.^32^

In Kenya, the expectation of long queues and poor-quality services was a barrier to linkage.^48^ Kenyan patients also feared being screened for HIV at facilities and avoided them.

Distance to a health facility influenced whether patients referred after screening would attend in rural Uganda,^36^ Kenya,^30^ Vietnam,^23^ and Indonesia.^25^ This was borne out in qualitative studies.^25,30^ In Malaysia, Rahmawati reflects on the difficulties that some elderly patients might have in getting to mobile clinics. Yet proximity to health services did not always improve uptake, Bovet et al report that in Dar Es Salaam, where services are near patients, uptake of appointments and treatment was very low.^35^

####  Knowledge and Beliefs

Studies in Kenya and Belize identified poor understanding of hypertension among those not seeking care after its detection^30,38^ coupled with doubt that medicine could alleviate symptoms, fear of taking it, and belief in witchcraft.^30^ Several other studies suggest that the lack of knowledge that hypertension is often symptomless impacts on uptake of services.^25,35,36,38,48^ The positive impact of education and awareness raising was described in 2 studies,^30,38^, although this did not reach statistical significance in the study from Belize.^38^

####  Trade-offs

Competing family and work responsibilities prevented linkage to care in rural Uganda,^36^ although transportation costs and difficulties were more frequently implicated. Obligations at work and home being prioritised against adhering to care were also cited qualitative research from Kenya, especially if services were of poor quality.^30^ Naanyu et al also describe concerns about being a drain on their own and their families’ resources.^48^

####  Social Relations and Traditions

Kotwani and Naanyu described how poor relations between health workers and patients in Uganda and Kenya were a barrier to seeking care following diagnosis.^36,48^ In Uganda, fear of being reprimanded for missing a scheduled appointment was cited by 26% of the 27 people interviewed who had failed to take up referral following community screening.^36^ Naanyu’s study implicated fear of harsh language by health workers.^30^ Rachlis et al described how good provider-patient relations were commonly reported to facilitate access to care following diagnosis.^30^ Rachlis et al also identified lack of partner support and inadequate social support as a barrier.^30^

###  Stage 3: Long-term Management

This stage covers patients who, having been diagnosed, are successfully referred into the system, received medication or a prescription, or were being followed up (or ‘linked’). Most studies of this stage of treatment addressed retention within the health system and adherence to medication (20 of 30).^19-30,33,34,37,40-42,45-47^ Barriers and facilitators were identified in all domains. That relating to health system resources was especially rich, with 21 studies reporting barriers related to financial, service delivery, medication, and geographical issues.

####  Demographics and Socio-Economic Factors

The evidence is extremely mixed. Four studies, from China and Malaysia, found that adherence was better among older patients,^19-21,24^ and women, who were also more likely to attend appointments.^19-21,24,30,40^ A qualitative study from Brazil attributed lower adherence and attendances by men to a macho culture,^40^ although another from Kenya reported how poor women had to prioritise domestic commitments and other calls on their finances.^30^ The association with education varied.^28,37^ The Brazilian study identified financial hardship as a barrier, with poorer patients less likely to seek continuing care after diagnosis or to use cheap proprietary remedies.^40^ In Malaysia, as before, ethnic differences were reported, with those of Malay or Chinese origin more likely to adhere to medication than those of Indian origin (odds ratio, OR: 1.68 [95% CI: 1.03–2.73] and OR: 2.64 [95% CI: 1.54–4.58 times], respectively).^24^ However, studies from Ghana and Namibia, found no significant association between age, sex, income, education/literacy, employment status, and adherence and hypertension control.^29,33^

####  Health Status and Co-morbidities

The relationship between poor health, or presence of co-morbidities, and effective follow up is inconclusive. Four reported that patients with fewer or no co-morbidities were less adherent to treatment and antihypertensive medications.^19,20,26,28^ In Malaysia, patients who also had diabetes were less likely to be adherent and have higher blood pressure who did not (OR: 1.74, 95% CI: 1.29-2.39)^24^ and a qualitative study in Colombia reported that some patients with multiple conditions considered hypertension to be unimportant.^42^ A qualitative study found that patients on multiple drug therapies stopped medication if they experienced adverse reactions and as advised by social networks.^27^ In Vietnam, the dropout rate was significantly higher among those with mild than severe hypertension (21.5% and 8.2% respectively, *P* < .01)^23^. Some personality types (stressed, strict, irritable, depressive or obsessive) were linked to poor adherence, including to dietary restrictions, in Iran,^45^ while a Brazilian study implicated depression, especially among those who lacked social support and where the service quality was poor.^40^ However, a study from Namibia found that patients with HIV/AIDs did not have lower adherence.^33^

The asymptomatic nature of hypertension was frequently invoked as an explanation for non-adherence.^22,25,34,37,40,45^ For example, a Chinese study reported how those with uncomplicated hypertension simply do not feel “sick”^22^ while, in Brazil, patients take medication according to how they feel, taking half doses or skipping doses.^40^ In Iran it was reported that some symptoms that patients associate with high blood pressure, such as numbness and blurred vision, increase adherence.^45^ Finally, Rahmawati and Bajorek describe lack of transport for elderly patients to mobile clinics as a barrier, although the authors consider that it was not possible to distinguish whether failure to attend the mobile clinic was due to lack of transport or the asymptomatic nature of the disease.^25^

####  Health System Resources and Processes

 Health systems barriers and facilitators to adherence and continuity of care could be found in all fifteen studies. As this was the richest domain, we sub-divided these factors into those related to financial, staffing and service delivery, medication, and geographic proximity.

*Financial: *Seven studies addressed this factor. In Colombia, barriers were created by gaps in coverage by the social security system and associated need for payment to doctors,^42^ while studies in China and Nigeria link care free at the point of use with better adherence.^19,34^ Two noted how those living in rural areas of Brazil and Colombia suffered a double disadvantage, as they were less likely to be covered by social security and the costs of medicines were higher.^40,42^ A study of the Brazil’s Farmácia Popular programme found major increases in continuity of treatment and adherence to medicines for non-communicable diseases when key essential medicines were provided for free, including through private sector pharmacies, while cost sharing by patients led to decreases.^41^ In Ethiopia, adherence to medications was 2 times (adjusted OR = 2.06, 95% CI = 1.13, 3.76) higher in respondents who obtain it at low or no cost compared to the rest.^26^ In Indonesia, free blood pressure checks were considered to facilitate access to care.^25^ However, Chinese studies reached different conclusions, with one finding greater adherence among those receiving public assistance^21^ while another found it to be greater among those paying fees.^19^

In Ghana, in a study where study participants were covered by the insurance scheme and had guaranteed access to antihypertensive medications from hospital pharmacies, 20% reported problems in obtaining them and this was a significant predictor or poor hypertension control (OR: 1.24, 95% CI: 1.02–1.49).^29^ Costs associated with purchasing medication was also cited as a barrier to care in Kenya.^30^

*Service delivery: *Six studies identified factors related to service delivery.^22,25,40,42,44-47^ The most consistent finding was that retention of patients and adherence to treatment were better where health facilities were accessible, with short waiting times, longer duration of appointments with physicians, and offering care that is perceived to be of higher quality. A study from Namibia noted that many people were aware when their next appointment is but not attending it, suggest a lack of ways to track the patients or send reminders, as well as providing incentives.^33^ One Chinese study found that an enhanced role for pharmacists (advising physicians of potential changes in medication and advising patients on adherence and life style), led to improved adherence.^22^ Conversely, the perceived lack of physicians, nurses, supplies and diagnostic equipment, high patient volumes and public providers lacking time to counsel on mediations and adapting lifestyles, transportation and cost were common barriers to routine check-ups in primary care facilities, with quality sometimes better than in the private sector.^44^ An absence of guidelines for blood pressure measurement is also a supply-side barrier, as are stock-outs of drugs in public facilities, with patients needing to seek their medication in private pharmacies, thus incurring costs for travel and medication.^44^ Long waiting times were identified as a barrier in 7 studies.^29,30,34,40,42,44-46^

Counterintuitively, in Ghana, blood pressure control was poorer among those treated at a tertiary facility in dedicated hypertension clinics, mainly in urban areas (OR: 2.47, 95% CI: 1.57–3.87) than in rural primary healthcare facilities which despite these facilities more accessible; this may be due to poorer conditions and longer waiting times.^29^ Longer duration of hypertension diagnosis also reduced the likelihood of successful control. A complex primary care intervention in Mexico involving a new cadre of community health workers, supply chain improvements, active case-finding, and education support for rural doctors did not lead to any significant improvement in blood pressure control among the population of Chiapas State.^43^

*Medication-specific issues:* In addition to problems associated with purchasing medication (see above), 4 studies reported on availability (or lack thereof) of medicine.^30,34,42,45^ Unsurprisingly, all reported lack of access, at health facilities,^42^ in pharmacies,^34^ and more generally^45^ as a barrier to adherence.

Twelve studies associated more complex medication regimes, polypharmacy with lower adherence,^19,21,22,24,28,29,34,40,42,45-47^ while 6 noted the adverse impact of side effects of medication on adherence,^30,34,37,40,45-47^ with one study from Malaysia finding that few patients were warned about them.^47^ Specifically, unclear or ambiguous explanation of regimens or polypharmacy by providers led to patients stopping or increasing medications (when feeling better or if concerned about side effects), researching and buying non-prescribed drugs.^27^ In some studies the use of traditional medicine was associated with poor adherence^28,45-47^ or described as an alternative to pharmaceuticals that were expensive or hard to find.^42^

*Geographical accessibility:* Five studies^19,30,34,42,46^ examined the role of proximity to health facilities. Four reported that patients living far away were less likely to attend but all were based on qualitative data.^30,34,42,46^ Other studies found that greater distance from a clinic^34^ or living in a different district than the hospital reduced adherence to medication.^19^ Support for costs of transport from family members was reported to facilitate continuity of care in Colombia.^42^

####  Knowledge and Beliefs

Fourteen studies identified limited knowledge about hypertension and its management as a barrier to adherence and retention,^22,24,26,27,30,33,34,37,40,42,45-47,49^ while one study found that although literacy about antihypertensive medication (as distinct from consequences of hypertension) was high (83% of patients), there was no significant association with adherence and attending appointments.^33^

Several themes emerged. One was that hypertension was viewed as a transient problem.^30,34,45^ Some Malaysian patients described not taking medication as prescribed because of a belief in their ability to control their blood pressure with physical activity, diet, and stress management.^47^ A Chinese study found patients who believed they had been cured.^22^ In Iran, while some believed that the body could recover by itself, others believed it was inherited and could not be treated.^45^ In Ethiopia, users with a favourable attitude — a possible proxy for trust — about antihypertensive treatment were ten times (adjusted OR = 9.88, 95% CI = 5.34, 18.27) more likely to be adherent than others.^26^

Another strand reflected broader perceptions of illness and disease. In several countries there was a belief that long term medication would cause damage to the body, especially the kidneys,^49^ or side effects^27^ while in Egypt,^37^ adherence was lower in those who believed that they were generally more likely than others to suffer misfortunes. In Ghana, perceptions that mainstream drugs were ineffective were associated with interrupting or terminating their treatment and substituting herbal medicines and alternative therapies, including spiritual healing, prayers, and fasting, seen as protective from witchcraft and spells. The impact of these beliefs was accentuated by the greater ease of obtaining affordable alternative therapies as well as trusted relationships with native providers and a general belief that ‘medication is unnecessary because ill-health is an act of God.’^27^ In Colombia and Brazil medication provided free of charge was sometimes considered inferior to that paid for. However, in Nigeria, faith in “orthodox medicines” (provided through the health system) was considered to improve adherence.^34^

One Malaysian study found a small, but statistically significant increase in adherence among patients with better knowledge of their medication (OR: 1.03, 95% CI: 1.01–1.04, *P* =.001)^24^, with similar findings from Egypt.^37^ However, health workers often lacked educational material and provided little information to patients.^30,46^ In Iran, patients identified information in the mass media as a source of information, although with mixed impact on adherence.

####  Trade-offs 

Eleven studies addressed trade-offs.^25,28,30,34,40,42,45-47^ In Iran, patients reported how being busy working (either outside the home or undertaking childcare) increased the likelihood of forgetting to take medication.^45^ However, in Malaysia, pressure from employers to be healthy, coupled with access to private providers facilitated adherence.^46^ In Namibia, missing appointments was very common (75% ever missing a scheduled clinic appointment) and in 60% of cases this was attributed to work commitments, despite being aware of the need for treatment,^33^ while in Ghana ‘preoccupation with routine work’ and sustaining livelihoods, including having to travel away from home, led to de-prioritisation of medication (often framed as ‘forgetfulness’).^27^

Seven studies addressed psychological factors.^25,28,30,40,42,45,47^ An unwillingness to defer gratification was identified as a barrier to adherence to treatment, including diet in Iran.^45^ Three studies identified low motivation or will-power as a barrier to retention.^25,30,42^ and in Indonesia the desire to be healthy was associated with enhanced continuity of care.^25^

####  Social Relationships and Traditions

Eleven studies addressed these issues.^25,30,33,34,37,40,42,45-47^ Relationships with families and friends could be either a facilitator or barrier to retention, with poor relationships with family members impacting negatively on adherence^30,34,47^; lifestyle modification^46^ and retention^30,42^ while in several studies family support encouraged adherence^30,34,45,47^ and retention in the system.^30,42^ For example, support from friends and/or relatives were found to be critical for adherence through encouragement to take medication and attend follow-up appointments in Namibia.^33^ There was little information on the role of local communities, although Shima et al reported how Indian patients in Malaysia were influenced by neighbours and friends when making decisions about adherence^47^ while, in Indonesia, peer support was an important motivator for patients to participate in a community-based programme for elderly patients.^25^

Seven studies addressed local cultural practices and traditions.^27,30,34,37,42,45,46^ Traditional practices could be a barrier to adherence^34,46^ and continuity of care.^46^ Thus in Ghana, there could be pressure on from peers, family, and relatives to choose traditional and herbal medicines, which were perceived to be safer, more effective and cheaper.^27^ The presence of fatty food at social events also made lifestyle changes difficult.^40,42,45^ In Nigeria, attitudes favouring smaller body size were linked to better adherence while in both Nigeria and Iran, those with stronger religious beliefs were more likely to be adherent^34,45^ but in Brazil fatty or salty foods are considered to give immense pleasure in later life and so difficult for older patients to forego.^40^

Where there were positive relationships between health workers, adherence was facilitated. Having a good patient –provider relationship increased the likelihood of adherence 4 times.^26^ In Nigeria, the approachability and social reputation of the doctor was linked to greater adherence.^34^ In Indonesia, community health workers encouraged continuity of care in a community by means of interactive discussions with older patients^25^ while in Kenya, good relationships were identified as increasing retention.^30^ However, a traditional hierarchical relationship between health workers and patients in some countries could act as a barrier, as in Brazil, where doctors adopted an authoritarian approach to older patients, who often lacked trust in those providing care.^40^

####  Barriers and Facilitators not Specific to a Single Domain

Two papers trace the patient’s journey overall.^31,39^ Most drivers were as in the other papers, with older women more likely to seek care and limited financial resources impeding continuity of care, while that those with co-morbidities were more likely to attend appointments. Health systems related barriers included high costs, medicine stock-outs, inaccessible facilities, and staff absences leading to low levels of satisfaction among patients.^31^ Care provided by nurses was considered to be a potential barrier in Kenya, depending on whether patients accepted them as primary care givers, or preferred alternative treatments reflecting beliefs in witchcraft.^31^

## Discussion

Control of hypertension remains poor everywhere but especially in LMICs.^2^ This systematic review examines barriers and facilitators along pathways followed by hypertensive patients — from first symptoms and entry into the system to treatment initiation and follow-up — that lead to poor control of their condition. We argue that a better understanding of these issues is an important step in achieving hypertension control, informing design of interventions. Thirty papers met the inclusion criteria. A conceptual framework with 6 domains was used to analyse the findings. The key findings are summarised in Box 1.

**Box 1.Key Findings**

- Patients with hypertension confront different barriers and facilitators on their journey through the health system, from diagnosis to treatment initiation to maintenance.
- The effects of barriers accumulate along the patient pathway and characteristics of the health system can reinforce or mitigate them.
- Knowledge and beliefs about hypertension are important at entry in the system but social relationships, traditions and presence of comorbidities become more important later.
- Patient pathways are non-linear and are best characterised as continual cycles of entry and re-entry into the system, as patients seek to accommodate their priorities with respect to health and life in general.
- More evidence is needed on the ways in which individual-, community- and health system-related barriers and facilitators interact, taking account of the patient’s perspective and their agency at each stage of the pathway if we are to design nuanced responses that improve hypertension control.

Several limitations must be acknowledged. The first relates to how access was conceptualised and what study designs were included. Most studies often reported 2 points of the care continuum, typically entry into the system and subsequent retention, and were not designed to capture intervening barriers and facilitators. Second, even those studies following the patient along the entire pathway often failed to differentiate the various stages. Third, studies often take a top-down perspective, defining treatment stages according to a predetermined clinical pathway or programme intervention, rather than reflecting the perspective of the patients, their needs and preferences. This was particularly the case for the follow-up stage during which patients may think their treatment has been completed; thus, the agency of the patients is often overlooked.

Despite these limitations, our findings show that different combinations of barriers appear to matter at each stage of the care pathway. At entry the key barriers and facilitators relate to how effectively patients are identified and how they learn about their condition (‘health systems resources and processes’ domain) — through primary healthcare services and/or community-based screening. The patient’s ‘knowledge and beliefs about hypertension’ domain is also key at this stage. For example, the asymptomatic nature of hypertension influences how the patient chooses to manage their condition, as would be expected, given the need for patients to recognise the importance of seeking care.^50^

 As patients move along the care pathway, they face an accumulating range of barriers. At the treatment (medication) initiation stage, most relate to ‘health systems resources and processes,’ pointing to the importance of a well-functioning health system. Co-morbidities act as a barrier (with some exceptions) as they complicate treatment. ‘Social relations and traditions’ also emerge at this stage and remain important in the follow-up stage.

 The largest number of studies address the follow-up/retention stage, which is where a wide array of issues come into play. Barriers and facilitators spanned all 6 domains, but the most important related to poorly resourced and managed health systems, ‘patient knowledge and beliefs’ and ‘social networks and relationships.’ Patients begin to make conscious ‘trade-offs’ of continuing treatment against fulfilling family and social roles, starting at the treatment stage but even more so at follow-up.

 While socio-economic characteristics are often a major issue in studies that examine only one point of the care pathway, particularly entry, overall they are often mitigated or overcome by characteristics of the health system and social networks, leading patients to make trade-offs between continuing treatment and meeting other priorities, such as work and family commitments.

Few studies sought to challenge the linearity of the pathway (from diagnosis to effective control), with the exception of Gabert et al who present it as a continuous cycle of entry and re-entry/remaining in the system.^44^ Most studies see the pathway as normative and singular, excluding the possibility of diverse trajectories or incomplete cycles constrained by factors within and beyond the health system. While the qualitative studies often involve an iterative analysis demonstrating the complexity of the interactions, this is often done only to interpret the findings and identify policy implications, rather than being integral to study design.

Most studies identify independently acting barriers and facilitators of effective care or hypertension control emerging along the patient pathway, but few explicitly demonstrate how these distinct factors interact or illustrate in what specific cases or contexts a set of enabling factors can help to overcome barriers. For example, living close to a health facility could be a facilitator of treatment, but not if family or social networks discourage access. More specifically, Atinga et al argue that factors cannot be viewed as a set of fixed causal sequences, but rather are interrelated, with each triggering a new cycle of behaviour (causal loops), while they show that the use of traditional and complementary medicines to treat hypertension could either result from or lead to perceptions that modern medication is ineffective and inappropriate.^27^

The review demonstrates how social relationships within and outside the health system are significant independent factors, but also mitigate other factors. Thus, patients observe or modify their behaviour according to social norms and advice from trusted networks on what is a serious condition, when to seek modern medicine to treat certain conditions and when to cope with family/traditional remedies which often contradict recommended treatment regimens.^46,48,51^ The relationship between providers and patients—reflecting the formal and informal treatment traditions—appears to be critical. However, information provided to patients is not always sufficient and understandable (eg, on how to take their medication, or what are the consequences of non-adherence to medication^33^).

 The included studies do not sufficiently capture the patient’s perspective and agency. Interestingly, while maintaining follow-up is important to clinicians, it may be less so from the point of view of the patients and their families. They may believe that making contact at the earlier stages of entry and treatment initiation is more important, while maintaining health afterwards can be done with their own resources and as time permits given other life commitments. Furthermore, fundamental beliefs about the nature and progression of disease and what constitutes a (high quality) treatment are central to care for hypertension, which may or may not be accepted as a largely asymptomatic condition, as an inherited disease, or as a part of the natural aging process. These perceptions are nested within a broader set of beliefs of how to manage life and how to reasonably balance ongoing treatment against other competing priorities, benefiting not only individuals but their families and social networks.

The study has important implications for policy. Stage-specific evidence about barriers to hypertension care that address the complexity of pathways and interplay of factors, can help to inform better targeted and effective hypertension control, which is consistent with emerging conceptions of ‘precision public health.’^52,53^ While measures suggested include tracking patients, setting-up a reminder system for clinic appointments,^33^ there is a recognition that interventions need to go beyond the health systems, for example to address the multiple competing demands on patients and their families.

There is also a need for health providers to adopt more people-centred treatment approaches that account for patients’ beliefs, values and norms in managing their condition, and to engage with the knowledge, treatment strategies and experience of medication by patients and their families,^54^ which has also been called as taking a ‘cultural competence therapeutic approach.’^27^ This review is part of a larger project that is consistent with these approaches and sees patients as active agents, determining how their treatment progresses, and gives them voice through the opportunities offered by mobile technology.^55^ Nevertheless, the balance of evidence suggests that a more comprehensive mix of measures is required: accessible health systems resources including information adapted to patients, but also addressing the structural causes of ill health and the trade-offs made. Health systems interventions and policies need to engage more closely with these domains, taking the long view.

Ultimately, the question is whether such an approach matters? This review is part of a lengthy programme of work we have undertaken over several years in which we have argued for such a patient-centred approach. This included 2 of the studies cited, in Malaysia and Colombia.^42,46^ These were used to design complex multi-faceted interventions adapted to each context and evaluated in a cluster randomised trial that achieved substantially improved control.^56^

 In conclusion, this review demonstrated that the patient pathway is influenced by a mix of individual-, community- and health system-related barriers and facilitators that act at different stages, often interacting. Many of the included studies were designed to capture some but not all of these, so the ensuing recommendations rarely reflect their complex interplay. More studies are needed that can distinguish between stages of care, acknowledge both the formal/normative and informal treatments and actors with which patients engage, and elucidate the many interacting factors that shape each patient’s journey. A more realistic conceptualisation of the patient pathway is important for more targeted policy recommendations, and our conceptual framework offers a useful tool to for further research on hypertension and other chronic conditions.

## Ethical issues

 Not applicable.

## Competing interests

 Authors declare that they have no competing interests.

## Authors’ contributions

 All authors made substantial contributions to the review design; the acquisition, analysis and interpretation of data; the drafting, critical revision and final approval of the work; and agree to be accountable for all aspects of the work.

## Funding

 This study is supported by a grant from the Wellcome Trust/Newton Fund-MRC Humanities & Social Science Collaborative Award scheme (200346/Z/15/Z). The funding source had no role in study design; in the collection, analysis or interpretation of data; in the writing of the article; or in the decision to submit it for publication.

## Authors’ affiliations

^1^Brown School, Washington University in St Louis, St Louis, MO, USA. ^2^Department of Global Health and Development, Faculty of Public Health and Policy, London School of Hygiene and Tropical Medicine, London, UK. ^3^Department of Health Services Research and Policy, Faculty of Public Health and Policy, London School of Hygiene and Tropical Medicine, London, UK.

## Supplementary file 1


Supplementary file 1 contains Table S1.
