# Supplementary material for: The Long and Winding Road: A Systematic Literature Review Conceptualising Pathways for Hypertension Care and Control in Low- and Middle-Income Countries
Source: Int J Health Policy Manag. 2020 Jul 18;11(3):257–68. doi: 10.34172/ijhpm.2020.105 (PMC9278472; doi:10.34172/ijhpm.2020.105)
Supplement: Supplementary file 1 — contains Table S1. [file ijhpm-11-257-s001.pdf]

Supplementary file 1

Table 1. Barriers and facilitators of patient progression in the health systems

Stage	Domain	Themes	Author and Country
Entry & diagnosis	Health systems resources & processes	- Patients from mass screening do not return for confirmation or diagnosis, little routine monitoring	Risso Gill et al., 2015 (Malaysia), Shima et al., 2014 (Malaysia)
		+ Adequate health systems resources, mass screening, routine screening, work-place screening	Risso-Gill et al., 2015 (Malaysia), Nations et al., 2011 (Brazil), Legido-Quigley et al 2015 (Colombia), Youssef and Moubarak, 2002 (Egypt)
	Knowledge and beliefs about hypertension	- Limited information and poor understanding of causes, symptoms; feeling well	Legido Quigley et al., 2015 (Colombia), Naanyu et al 2016 (Kenya), Risso Gill et al 2015 (Malaysia), Gaber et al., 2017 (India)
		+ Having symptoms	Legido Quigley et al., 2015 (Colombia), Risso Gill et al., 2015 (Malaysia), Shima et al., 2014 (Malaysia)
Progressing through the system: initiation of treatment, first referral	Demographics and socio-economic factors	- Poverty, work responsibilities and need to balance care needs with other daily needs	Bovet et al., 2008 (Tanzania), Kotwani et al., 2014 (Uganda), Naanyu et al., 2016 (Kenya), Rachlis et al., 2016 (Kenya)
		+ More advanced age	Bovet, 2001 (Tanzania), Chung, 2005 (Belize), Kotwa et al., 2014 (Uganda), Nguyen et al., 2011 (Vietnam)
		+/- Being employed, higher education, being female	Chung et al., 2005 (Belize), Kotwani et al., 2014 (Uganda), Naanyu et al., 2016 (Kenya)

	Health status and co morbidity	- History of other chronic diseases, behavioural risk factors, milder hypertension, forgetfulness, poor motivation, lack of symptoms	Nguyen et al., 2011 (Vietnam), Rachlis et al., 2016 (Kenya), Bovet et al., 2008 (Tanzania), Kotwani et al (Uganda), Naanyu et al, 2016 (Kenya), Rachlis et al 2016 (Kenya), Rahmawati & Bajorek, 2015 (Indonesia
		+ Severe hypertension, current tobacco use, higher initial blood pressure, personal history of hypertension or CVD, worsening health status, being overweight, personal initiative	Bovet et al., 2008 (Tanzania), Chung et al., 2005 (Belize), Nguyen et al., 2011 (Vietnam), Rachlis et al., 2016 (Kenya), Kotwani et al (Uganda)
		+/- Alcohol use, family history of CVD or hypertension, worsening health status/ severe hypertension	Naanyu et al., 2016 (Kenya), Rachlis et al., 2016 (Kenya), Kotwani et al., 2014 (Uganda), Nguyen et al. 2011 (Vietnam)
	Health systems resources and processes	- Costs associated with care and treatment, treatment and traditional remedies available outside health system, poor quality of care, lack of staff and/or specialised treatment, long queues, longer distance and higher cost of transport, poor linkage following community level entry	Bovet et al., 2008 (Tanzania), Kotwani et al., 2014 (Uganda), Naanyu et al., 2016 (Kenya), Nguyen et al., 2011 (Vietnam), Rachlis et al., 2016 (Kenya), Rahmawati & Bajorek, 2015 (Indonesia), Risso Gill et al., 2015 (Malaysia), Shima et al., 2014 (Malaysia), Subramanian et al. 2018 (Kenya)
		+ Availability and training of healthcare providers, accessible of clinic, Comprehensive counselling and appropriate referral	Nguyen et al., 2011 (Vietnam), Rachlis et al., 2016 (Kenya), Kotwani et al., 2014 (Uganda)

		+/- Availability of traditional remedies outside the health system	Naanyu et al., 2016 (Kenya), Rachlis et al., 2016 (Kenya), Bovet et al., 2008 (Tanzania)
	Knowledge and beliefs about hypertension	- Lack of knowledge and poor understanding about hypertension (especially symptomlessness), stigma	Bovet et al., 2008 (Tanzania) Chung et al., 2005, (Belize) Kotwani et al., 2014, (Uganda), Naanyu et al. 2016, (Kenya) Rahmawati and Bajorek, 2015, (Indonesia), Rachlis et al., 2016 (Kenya), Chung et al., 2015 (Belize)
		+ History of hypertension, community awareness, sensitisation/ education	Chung et al., 2015 (Belize), Rachlis et al., 2016 (Keny
	Social relations and traditions	- Family responsibilities, fear of being screened for stigmatised disease (HIV), lack of social or family support, poor relationships between patient and health worker	Kotwani et al., 2014 (Uganda), Naanyu et al., 2016 (Kenya), Rachlis et al., 2016 (Kenya)
		+ Good provider patient relationships, concerns about family responsibilities, peer, family and social support	Rachlis et al., 2016 (Kenya)
	Trade-offs	- Balancing care needs with other daily demands including work and home	Rachlis et al., 2016 (Kenya), Naanyu et al., 2016 (Kenya), Kotwani et al., 2014 (Uganda)

Follow up stage/ retention in the system	Demographics and socio-economic factors	+ / - Older age, higher education, gender, lower socio-economic status	Harries, 2005 (Ghana), Legido Quigley et al., 2015 (Colombia), Nashilongo et al., 2017 (Namibia), Natio[illegible] et al., 2011 (Brazil), Rachlis et al., 2016 (Kenya), Ram[illegible] et al., 2012 (Malaysia), Sarfo et al., 2018 (Ghana), Youssef & Moubarak, 2002 (Egypt), Wong et al., 200[illegible] (Hong Kong), Wong et al., 2011 (Hong Kong), Wong [illegible] al. 2015 (Hong Kong)
	Health status and co-morbidity	+/- Comorbidities, Higher blood pressure, severity of hypertension	Atinga et al., 2018 (Ghana), Harries et al., 2005 (Ghana), Legido Quigley et al., 2015 (Colombia), Mekonnen et al., 2017 (Ethiopia), Nashilongo et al., 2017 (Namibia), Nations et al., 2011 (Brazil), Nayeri e[illegible] al., 2015 (Iran), Nguyen et al., 2011 (Vietnam), Ramli [illegible] al., 2012 (Malaysia), Wong et al., 2009 (Hong Kong), Wong et al., 2011 (Hong Kong)
		- Lack of symptoms	Nations et al., 2011 (Brazil), Nayeri et al., 2015 (Iran), Odusola et al., 2014 (Nigeria), Rahmawati & Bajorek, 2015 (Indonesia), Youssef and Moubarak, 2002 (Egyp[illegible] Zhao et al., 2012 (China)
		+ Feeling ill or improving under exercise program	Nayeri et al., 2015 (Iran), Rahmawati & Bajorek, 201[illegible] (Indonesia)

	Health systems resources & processes	+/- Costs associated with receiving care and medicine	Emmerick et al., 2017 (Brazil), Legido Quigley et al., 2015 (Colombia), Mekonnen et al., 2017 (Ethiopia), Nations et al., 2011 (Brazil), Odusola et al., 2014 (Nigeria), Rachlis et al., 2016 (Kenya), Rahmawati & Bajorek, 2015 (Indonesia), Wong et al., 2009 (Hong Kong), Wong et al., 2015 (Hong Kong), Sarfo et al., 2018 (Ghana)
		- Complex medication regimes, polypharmacy, side effects, use of traditional medicine	Atinga et al., 2018 (Ghana), Harries et al., 2005 (Ghana), Legido Quigley et al., 2015 (Colombia), Nations et al., 2011 (Brazil), Nayeri et al., 2015 (Iran), Odusola et al., 2014 (Nigeria), Rachlis et al., 2016 (Kenya), Ramli et al., 2012 (Malaysia), Risso-Gill et a[illegible] 2015 (Malaysia), Sarfo et al., 2018 (Ghana), Shima et al., 2014 (Malaysia), Youssef & Moubarak, 2002 (Egypt), Wong et al 2009 (Hong Kong), Wong et al 2015 (Hong Kong), Zhao et al., 2012 (China)
		- Poor quality of service (lack of follow-up, tracing of patients, reminders of appointments, lack of explanation of treatment, short, infrequent visits, slow service and lack of appointments, visiting multiple clinics, lack of specialist services –	Atinga et al 2018 (Ghana), Gabert et al., 2017 (India), Legido Quigley et al., 2015 (Colombia), Nashilongo e[illegible] al., 2017 (Namibia), Nations et al., 2011 (Brazil), Nay[illegible] et al., 2015 (Iran), Odusola et al., 2014 (Nigeria), Rachlis et al., 2016 (Kenya), Rahmawati & Bajorek, 2015 (Indonesia), Risso Gill et al., 2015 (Malaysia),

		especially related to patients being seen by general practitioners or in accident and emergency settings, lack of medicine), distance to the health centre or pharmacy.	Sarfo et al., 2018 (Ghana), Shima et al., 2014 (Malaysia), Wong et al., 2009 (Hong Kong), Wong et al., 2015 (Hong Kong), Zhao et al 2012 (China)
		+ Pharmacist involvement or private sector providers, good relationships between providers and patients	Atinga et al., 2018 (Ghana), Gabert et al., 2017 (India) Mekonnen et al., 2017 (Ethiopia), Risso Gill et al., 201 (Malaysia), Wong et al., 2015 (Hong Kong), Zhao et a 2012 (China)
	Knowledge and beliefs about hypertension	+ Good knowledge about hypertension or appropriate food to eat and weight loss, favourable attitude, faith in treatment or orthodox/ biomedicine medicines	Legido Quigley et al., 2015 (Colombia), Mekonnen et al., 2017 (Ethiopia), Nayeri et al., 2015 (Iran), Odusola et al., 2014 (Nigeria), Rachlis et al., 2016 (Kenya), Ramli et al., 2012 (Malaysia), Youssef & Moubarak, 2002 (Egypt)
		- Poor knowledge about hypertension, Belief that the body can recover by itself, that medication brings cure, that high blood pressure is part of being old or is transient, that medicines damage the body. Belief in witchcraft and spells	Atinga et al., 2018 (Ghana), Legido Quigley et al., 201 (Colombia), Manto et al 2018, Nayeri et al., 2015 (Iran Odusola et al., 2014 (Nigeria), Rachlis et al., 2016 (Kenya), Risso Gill et al., 2015 (Malaysia), Shima et a 2014 (Malaysia), Zhao et al., 2012 (China), Manto et a 2018 (Cameroon)

		+ pressure of employers to be healthy, personal initiative, desire to be healthy	Legido Quigley et al., 2015 (Colombia), Rachlis et al., 2016 (Kenya), Rahmawati & Bajorek, 2015 (Indonesi[illegible] Risso Gill et al., 2015 (Malaysia)
	Trade offs	- Pressures of paid work, domestic work, unwillingness to defer gratification, low motivation or will power	Nayeri et al. 2015 (Iran), Nashilongo et al., 2017 (Namibia), Atinga et al.,2018 (Ghana), Legido-Quigle[illegible] et al., 2015 (Colombia) Rachlis et al., 2016 (Kenya), Rahmawati and Bajorek, 2015 (Indonesia)
		+ Employer pressures on employee to be healthy, personal desire to be health	Rahmawati and Bajorek, 2015 (Indonesia), Risso-Gill [illegible] al. 2015 (Malaysia)
	Social relations and traditions	- Lack of social support, misinformation from community peers and the media, poor relationships and communication between patients and health staff,	Legido Quigley et al., 2015 (Colombia), Nations et al. 2011 (Brazil), Nayeri et al., 2015 (Iran), Odusola et al. 2014 (Nigeria), Rachlis et al., 2016 (Kenya), Risso Gi[illegible] et al., 2015 (Malaysia), Rahmawati and Bajorek 2015 (Indonesia), Shima et al., 2014 (Malaysia)
		+ Supportive relatives and communities, good relationships between patients and staff, good social reputation of doctor	Legido- Quigley et al 2015 (Colombia) Nashilongo et al., 2017 (Namibia), Nayeri et al., 2015 (Iran), Odusol[illegible] et al., 2014 (Nigeria), Rachlis et al., 2016 (Kenya), Rahmawati & Bajorek, 2015 (Indonesia), Shima et al., 2014 (Malaysia)
		+/- Local cultural practices and traditions	Atinga et al., 2018 (Ghana), Legido Quigley et al., 201[illegible] (Colombia), Nayeri et al., 2015 (Iran), Nations et al 2011., (Brazil), Odusola et al., 2014 (Nigeria), Rachlis[illegible]

			et al., 2016 (Kenya), Risso Gill et al., 2015 (Malaysia) Youssef & Moubarak, 2002 (Egypt)
Barriers and facilitators not specific to a single stage	Demographics and socio-economic factors	+ Older age, being female	Ferreira et al., 2015 (Brazil)
		- Poor financial resources	Vedanthan et al., 2016 (Kenya)
	Health status and co-morbidity	+ being in poor health, being in good health, having co-morbidities (cancer, heart disease, diabetes)	Ferreira et al., 2015 (Brazil)
		- Costs of medication, consultation, lab tests, transport, difficulty of reaching facility, fear of stigma when care is provided by HIV clinic, poor timekeeping among staff, stock outs of medicines, dislike of nurse led services	Vedanthan et al., 2016 (Kenya)
	Health systems resources & processes	+ Being covered by health insurance, liking nurse led services	Ferreira et al., 2015 (Brazil), Vedanthan et al., 2016 (Kenya)
		- No symptoms, belief that symptoms relate to witchcraft not hypertension, lack of knowledge and community awareness of hypertension	Vedanthan et al., 2016 (Kenya)
		+ Fear of mortality	Vedanthan et al., 2016 (Kenya)

	Knowledge and beliefs about hypertension	- Lack of patient satisfaction	Vedanthan et al., 2016 (Kenya)
	Social relations and traditions	+ Strong community support, patient satisfaction and trust between patients and nurses	Vedanthan et al., 2016 (Kenya)

+ facilitator of patient progression; - barrier to patient progression
